# The impact of COVID-19 lockdown - case report

**DOI:** 10.1192/j.eurpsy.2021.1750

**Published:** 2021-08-13

**Authors:** B. Jesus, J. Martins Correia, S. Freitas Ramos, D. Cruz E Sousa, M.I. Fonseca Marinho Vaz Soares

**Affiliations:** 1 Department Of Psychiatry And Mental Health, Local Health Unit of Guarda, Guarda, Portugal; 2 Psychiatry And Mental Health, Hospital, Guarda, Portugal

**Keywords:** COVID-19, Anxiety, lockdown, mental health

## Abstract

**Introduction:**

In early 2020, governments started to implement different forms of public health measures, from physical distancing recommendations, to stay-at-home orders, to limit the propagation of COVID-19. Here we report the case of a 41-year-old woman, with a diagnosis of panic disorder. During the end of the lockdown, the patient presented psychopathological worsening, from her fear of Covid-19 infection, stemming from a heart failure disease and concerns regarding the hygiene and safety measures of those around her.

**Objectives:**

Presentation of a clinical vignette.

**Methods:**

Selection and analisis of clinical case and review of the literature using PubMed database.

**Results:**

The COVID-19 pandemic and the measures adopted to prevent the spread of the disease had a huge impact on a personal, social, and economic level for the world population. The rise of fear and anxiety among people due to uncertainty about the disease are coupled with essential yet disruptive measures such as lockdowns and quarantines. The chronically ill population are especially vulnerable during such circumstances and require addressing their physical health and any psychological difficulties they might experience, being at higher risk of suffering physically from the pandemic’s disease as well as psychologically from the implemented countermeasures.

**Conclusions:**

This vignette provides a case where a person’s psychiatric conditions are worsened due to the end of a pandemic lockdown, rather than the lockdown itself. Additional work should aim at comparing the experiences of the different countries affected by the pandemic in order to understand the size of the psychological impact, the potential risk and protective factors.

**Disclosure:**

No significant relationships.

